# Equity by Design: Redefining the Architecture of Dental Research in a Global Context

**DOI:** 10.7759/cureus.108931

**Published:** 2026-05-15

**Authors:** Vivek Sharma, Uttam Shetty, Viraj Kharkar, Swati Dhone, Saudamini More, Shreyas Neelkanthan

**Affiliations:** 1 Department of Periodontology, Bharati Vidyapeeth (Deemed to be University) Dental College and Hospital, Navi Mumbai, IND; 2 Department of Prosthodontics, Bharati Vidyapeeth (Deemed to be University) Dental College and Hospital, Navi Mumbai, IND; 3 Department of Oral and Maxillofacial Surgery, Bharati Vidyapeeth (Deemed to be University) Dental College and Hospital, Navi Mumbai, IND; 4 Department of Paediatric and Preventive Dentistry, Bharati Vidyapeeth (Deemed to be University) Dental College and Hospital, Navi Mumbai, IND; 5 Department of Public Health Dentistry, Bharati Vidyapeeth (Deemed to be University) Dental College and Hospital, Navi Mumbai, IND

**Keywords:** equity-oriented research, global oral health, health equity, oral health disparities, social determinants of health

## Abstract

There are significant numbers of people who suffer from oral diseases worldwide; however, dental research remains behind in embracing health equity principles in its practices. Health equity means the existence of a fair and just lack of difference in health status between populations and opportunities for all people to achieve the maximum degree of health possible. Besides being an ethical issue, health equity is also a necessity to guarantee the objectivity, inclusion, and relevance of scientific research. This narrative literature review seeks to discuss the need for health equity to be included at all stages of dental research. Hypothesis formation, study design, research practice, data analysis, and dissemination of results are the topics discussed in the review. The global impact of diseases of the mouth on public health will be described, as well as the factors behind the disparity of oral health status around the world. Also, the existence of epistemological issues within the methodology used for dental research will be highlighted, possibly leading to biased and non-inclusive scientific knowledge creation. Finally, new perspectives and methodologies are introduced, allowing researchers to rethink dental research and adapt it to society's needs.

## Introduction and background

Health equity as a scientific imperative

Dental research, like all biomedical research, cannot be considered value-free. The issues selected, the populations studied, the endpoints measured, and even the interpretation of findings are inevitably influenced by prevailing societal values. When the interests of society do not include groups that bear the brunt of oral disease, a skewed body of scientific knowledge results. The work may be rigorous, precise, and methodologically advanced, but still fall short when it comes to guiding the necessary interventions. Although such efforts may be scientifically rigorous, they may still have limited impact in developing interventions that are both effective and equitable. The importance of this issue becomes apparent when one considers the disparity of oral diseases that continues to exist worldwide [1]. Indeed, the persistent influence of social, economic, and structural inequalities on oral health outcomes is what makes the challenge of health equity in dental research so significant.

The problem of the global burden of oral diseases provides the evidence-based background for the question raised. According to the Global Burden of Disease (GBD) Study 2021, untreated dental caries in permanent teeth remains one of the most prevalent health conditions globally, while more than one billion adults are affected by severe periodontal disease. Edentulism plays a significant role in disability-adjusted life years (DALYs) across populations worldwide [1]. In comparison with the year 1990, the prevalence of oral diseases has increased considerably because of an increase in population, aging, and a low level of prevention care from 1990 to 2021. According to current predictions, the same trend may be observed up to the year 2050 unless structural changes occur in the field of oral disease prevention care [2]. This burden is not randomly distributed across populations but is rather consistent and follows certain patterns based on economic underdevelopment, manifested through poverty, lack of education, geographical remoteness, and insufficient access to the healthcare services provided. One in four individuals affected by oral disorders lives in low- and middle-income countries (LMICs), while 88% of global oral health expenditures are concentrated in countries inhabited by only 22.5% of the world's population [3]. 

It is precisely this inverse relation between where the disease lies and where the resources go that characterizes global inequities in oral health. Global oral health research should likewise take this as its key problem. The field has certainly played an important role in advancing the science and technology of prevention, treatment, and care for oral diseases. The area has not been nearly as successful in trying to understand and deal with the fundamental structural and socioeconomic issues which lead to the higher disease rates suffered by poor and vulnerable groups in the world [3]. Health equity demands that the balance between these contributions be redressed, with equity becoming part of what defines research rather than something added on.

The following sections address the argument that health equity needs to become an essential component of global oral health research at each stage of the research process, from beginning to end. The first section addresses the global structure of oral health inequities. The next section discusses the conceptual and methodological failings of the current global oral health research paradigm.

## Review

The global architecture of oral health inequity

The Socioeconomic Gradient: A Universal Pattern

One of the most well-documented phenomena in oral health literature is the socioeconomic gradient phenomenon, whereby the prevalence and severity of oral diseases tend to rise as people's socioeconomic standing becomes lower. According to the World Health Organization (WHO), there exists an exceptionally strong and clear link between socioeconomic standing, determined using income, occupation, and educational attainment variables, and the prevalence and severity of oral diseases, regardless of the age group under study (children through elderly individuals) and irrespective of whether it is high-, middle-, or low-income countries [4]. This is not simply a statistical correlation, but a reflection of how socioeconomic stratification influences biological outcomes.

It works on multiple pathways. For example, poverty makes the pursuit of dentistry services and the use of fluoride products more difficult [4]. Low education levels make people less knowledgeable and less able to navigate complicated healthcare systems. In disadvantaged environments, people are limited to locations where there are poor drinking water facilities, inadequate diets, and poor dental services. Lastly, psychosocial stress, which is socially influenced, affects the immune system of the body and causes people to adopt actions that increase caries and gingival diseases. No single pathway can fully explain the impact of the gradient [4]. All should be considered at once because all of them have social and political solutions that can be applied to alter them. The research that focuses on a single pathway must underestimate the impact of the structural forces on oral health outcomes.

Studies utilizing several national databases and cross-national research designs have demonstrated that income inequality, as reflected by the Gini coefficient, independently predicts caries experience; it would thus appear that oral health disparities are associated with relative rather than absolute deprivation in populations [5]. In stratified societies where a great deal of economic disparity exists, oral diseases serve as an index of social status, occurring in abundance at the lower end of the social hierarchy and conferring additional health risks that are exceedingly difficult to address once established [4,5].

The global-local divide: between-country and within-country inequity

Oral health inequities occur at the global level among countries of varying levels of income and also at the national level, geographically, socioeconomically, and socially. At the global level, the gap is pronounced. Countries that have publicly funded dental insurance coverage have significantly reduced socioeconomic inequalities in the use of dental care compared to countries without such systems [3]. In many developing nations, access to dental care is virtually impossible for most citizens, as seen in a scoping study conducted on 27 low-income nations where there were very poor dental care use and high out-of-pocket costs and where families from poorer socioeconomic backgrounds who used out-of-pocket payments to pay for necessary oral healthcare were more prone to catastrophic health expenses than families from wealthier socioeconomic groups [3]. Some of these nations had surveys showing tooth decay in the majority of adult patients and signs of periodontal diseases in almost all the adult patients surveyed while struggling with a constant shortage of dental practitioners [6].

Inequity, however, persists even within wealthier nations. In the socially disadvantaged groups such as those without shelter or stable homes, those in the incarceration system, those diagnosed with severe mental conditions, elderly individuals residing in assisted living facilities, and those in remote and rural locations, there are greater incidences of oral diseases, increased visits to dental offices for emergencies, and significantly lesser instances of preventive services when compared to the average citizen [7]. The findings from a systematic review on the oral health status of these groups revealed elevated incidences of carious lesions and periodontitis, a tendency toward visiting dental offices based on pain instead of preventive purposes, and the scarcity of research data on some of these social minorities, an omission from the scientific literature that in itself constitutes inequity [7]. Not being accounted for in studies does not render them invisible or non-existent; rather, it perpetuates the state of inequity that continues unabated.

There is no disconnect between global and local issues in oral health disparities; rather, there are links connecting them due to common structural reasons. The way globalization creates greater access to cariogenic foods in LMIC markets, the history of colonial exploitation in the underdevelopment of their healthcare infrastructures, and the fact that research is conducted and funded more in rich countries than elsewhere link together the between-country disparities seen globally to those that exist within countries as shown by national studies [3].

Commercial determinants: the corporate architecture of oral disease

The most critical yet poorly studied determinants of oral health inequalities include the role played by the corporate sector in creating and influencing the environmental conditions that shape risks of oral diseases on the population level [8]. The Federation Dentaire Internationale (FDI) World Dental Federation (WDF) updated its position statement regarding social and commercial determinants of oral health in 2023 and highlighted that certain corporate practices contribute to an increased burden of noncommunicable diseases including oral diseases [7,8]. Thus, the statement acknowledges the significance of implementing upstream policies with a common risk factor (CFR) approach for reducing the prevalence of oral diseases and inequalities [8].

The Global Oral Health Action Plan (GOHAP) 2023-2030 recognizes the necessity of addressing the impact of commercial determinants as one of the main objectives and defines commercial determinants of oral health as health problems caused by corporate activity such as the design of products, marketing practices, and lobbying of policies [9]. The 2024 Bangkok Declaration of the United Nations on oral health was the first political document on oral health to highlight the significance of addressing the problem [9]. Scientific literature demonstrates a link between the aggressive marketing strategies used to promote sugared drinks and highly processed foods globally and the growing burden of caries among children in LMICs [8,9].

Corporate lobbying against policies that would decrease caries by reducing the consumption of cariogenic foods such as sugar taxes or advertising bans is a clear obstruction to the creation of a positive policy environment for dental research [10]. Research focused strictly on the behavior-based factors affecting caries risk but failing to explore the commercial determinants behind dietary conditions will inevitably misidentify causality and misguide intervention efforts. For dental researchers uninterested in the literature on commercial determinants, their maps miss key influences on the terrain [9].

The research funding paradox

One structural aspect of global inequality in oral health that can be considered internal to the research enterprise involves the disparity between the location of the burden of illness and the funding of research related to it. Research has shown that there is a consistent pattern of undersupporting research on oral health where the burden of disease is largest, while those areas of the world most well-supported by research on oral health are countries that experience less severe oral health issues [10]. This has led to a body of dental knowledge that is poorly representative geographically and sociologically.

Among the countries that make up the WHO African Region, which has seen the biggest increase in oral diseases among all WHO regions in the last 30 years, more than half lack an oral health policy, while 70% allocate less than $1 annually per person for oral healthcare [11]. Research facilities in such countries are often poor, hampering the ability to gather country-specific data, engage in systematic monitoring of oral health, and provide adequate training to investigators that will lead to the development of local research capacity [10]. Without appropriate research capacity in place, health policy will necessarily rely on evidence generated elsewhere, which will be difficult, if not impossible, to tailor to local realities in terms of epidemiology, organization of health systems, culture, and finances.

Why health equity must be embedded in research design

Moving Beyond Disparity Documentation

Disparities in prevalence, severity, and lack of treatment have been shown by cross-sectional studies and epidemiological analysis to characterize oral diseases in disadvantaged communities for many years now [12]. This has formed the basis of the empirical case for equity as well as contributing to the political pressure leading to international commitments like the GOHAP. However, without an understanding of the causes of these disparities and without acting upon that understanding to affect structural change, inequity persists while creating an impression of engagement with science [12].

It has been contended by Tsakos et al. that it is necessary to transcend simply documenting inequities and instead focus on theory building, identifying causality, and setting research agendas that have the potential to alter the way in which oral health equity is distributed [12]. Sticking to documentation will serve the purpose of making inequities an integral part of the picture, which means that these become accepted, and even naturalized, as part of what the researcher encounters. Moreover, such an approach makes it easy to offer explanations regarding disparities, based upon the characteristics of those individuals who face such disadvantages and their personal and cultural behaviors [10]. However, the consequences of doing so are that interventions end up being designed for those individuals and that the social and historical structures underlying those behaviors remain untouched.

This trend was described by Watt as the most predominant type of preventive effort in dentistry, characterized by a very narrow "downstream" perspective [1], which involves modifying the behavior of high-risk persons and which not only has failed to alleviate the issue of oral health inequities in populations but could have also exacerbated the gap because the very populations who will have the most ability to change their behavior are the ones that are most privileged [13]. The paradigm needs to be changed to an upstream intervention that deals with the structural factors that inhibit people from sustaining healthy behaviors. Research that cannot produce knowledge that can change this paradigm is merely documenting the problem.

The CFR approach and social determinants integration

The CRF approach, which has been highly significant in global policies relating to oral health for over two decades, is based on the observation that oral diseases have many common risk behaviors including sugar intake, smoking, drinking, and poor self-care practices along with other noncommunicable diseases [14]. The CRF approach is more effective compared to disease-specific interventions focused on oral diseases alone due to its integrated approach towards dealing with these common risk factors. Sheiham and Watt developed a sound rationale for adopting the CRF approach, while the WHO policy later adopted it as the guiding principle for integrating oral health into the larger realm of noncommunicable diseases [14].

But Watt and Sheiham later contended that dentists and public health practitioners implementing oral health promotion have taken a myopic view of the CRF approach that focuses almost exclusively on behavioral factors at the expense of social factors which affect individuals' risk for all kinds of chronic diseases [15]. A purely behavioral model will be ineffective in reducing oral health disparities, and indeed could increase them, since health behaviors are themselves socially determined. The CRF approach modified within a social determinants framework will refocus efforts from altering behaviors to addressing the structural circumstances of income disparity, nutrition, education, shelter, and healthcare access which determine what behaviors are possible [14,15]. Science based on such a model raises different questions and requires different methods from those used within a behavioral science framework.

The representational gap in dental clinical research

Clinical trials provide the scientific base for evidence-based dentistry. However, the subjects involved in dental clinical trials were often selected based on accessibility, which usually meant students enrolled at universities whose social status allowed them to take part in researches because of their economic background, proximity to the location where the experiment takes place, literacy level, and ability to lead an even and stable life [16]. These subjects may not be representative of all other possible individuals, but the results are used anyway worldwide.

The results will have low validity and generalizability, and there is even the possibility that they might widen the disparity between the already disadvantaged population groups [16]. Interventions found to be useful when used on populations having abundant resources and living in stable environments will not work when applied to populations that are structurally disadvantaged simply because the factors that affect their reaction to the intervention may have been overlooked during the initial study. Improving the diversity and representativeness of dental clinical research is a matter of ethics as well as science.

Intersectionality and the limits of single-axis analysis

As per the WHO policy, however, social determinants are divided into structural determinants, which are the political, economic, and social systems responsible for stratification, and intermediary determinants, which are the conditions in terms of the material context, psychosocial influences, and behavior resulting from the structural arrangements [4]. This is important in the design of researches because addressing only the intermediary determinants while leaving the underlying structural factors intact will result in temporary improvements overshadowed by structural realities.

Another refinement of theory, gaining increasing significance in studies on health equity, involves the idea of intersectionality that individuals inhabit multiple and intersecting social locations within relations of stratification with regard to factors, such as class, gender, geography, age, disability, and others, and that these locations operate in complex and interactive fashion which cannot be understood merely through analysis of any single factor [16]. For example, an older individual in poor health who is also poorly educated and resides in a rural area of a poorer country faces obstacles to oral healthcare which are not merely additive; their interactions generate a particular constellation of problems. Designs for research that assume social factors to operate independently and additively will necessarily understate the weight of disadvantage borne by those who fall at the intersections of many kinds of disadvantage and will design interventions appropriate only to lesser degrees of disadvantage [12,16].

Reorienting research practice: frameworks for equity

Community Engagement as Epistemic Justice

The most profound manifestation of inequity within dental research lies in its epistemic form, whereby the marginalized communities are denied access to the knowledge-generation process about themselves [3]. Traditional frameworks for conducting research have relied on the assumption that communities are merely data providers who can conduct tests, surveys, and biological samples without any contribution to governance, analysis, and translation, which is conducted solely by external researchers [3]. This approach is both unethical and epistemologically restrictive because the communities possess unique knowledge about the socio-historical, ecological, and relational influences that determine their health status.

Community-based participatory research (CBPR) is arguably the most advanced methodological approach within the field that addresses the above problem. CBPR is premised on the notion that the community members, organization leaders, and academics should be equal partners throughout the entire research process, from setting the research questions, designing the study, data collection, data analysis, data interpretation, to disseminating the results [17,18]. Evidence from systematic reviews suggests that increased involvement of communities in health-related research leads to increased effects of programs that affect health through the social determinants of health [17]. The principle is universally applicable: regardless of whether the community lives in a poor rural or rich urban environment, its involvement leads to more relevant research, easier implementation, and sustainable improvements in health.

Community engagement is not a tool to use in an otherwise typical research process but rather represents a different kind of relationship between academics and community members altogether. There are three main ideas which recur throughout the literature on community-based research: trust, time, and co-design [18]. Trust has to be built up through ongoing presence and accountability and cannot be assumed. Time recognizes that building partnerships takes more than can be fit into traditional grant periods; institutions will need to change to accommodate community-based research practices [17]. Co-design recognizes that questions, methodology, and dissemination strategies should all be decided on collaboratively rather than asking the community to approve of what academics already chose.

Research capacity as a global equity requirement

Who creates dental knowledge raises issues of equity that are just as significant as what is created. Research that is conducted by investigators from wealthy nations will create questions, methods, and conclusions that are based on those contexts even if its intention is to be global. Hence, the creation of oral health research capability in LMICs cannot be seen as a peripheral development project. Instead, it must be understood as a necessity for a research process that truly represents the interests of those communities that suffer most from oral disease [19]. The global oral health workforce shows a strong relationship between the workforce density of oral health personnel and gross domestic product per capita. This demonstrates the same pattern of maldistribution of resources and capabilities that has been seen previously in research funding and disease burden [19]. The creation of global research partnerships and fellowship programs aimed at creating oral health research capability in low-income countries represents an investment in the epistemic sovereignty of these countries. The objective to reach a global target under GOHAP whereby 50% of the nations should have an agenda for oral health research targeting public health by 2030 recognizes this importance and brings it under international obligation [11].

Dissemination of the findings is equally important. Due to the high number of English-language publications of high impact, and the fact that subscriptions are too costly for low-income countries, the flow of information goes one way, from rich countries to poorer ones, but does not necessarily go the other way [10,19]. Publication through open-access journals, translating and disseminating key findings, and involving decision-makers and healthcare providers in the relevant countries are key to a truly global system of research.

Methodological plurality and upstream evidence

The creation of evidence that could be used to bring about structural change will require methodologies different from the ones that have been dominant in dental research so far [12]. While the clinical trial can provide high-quality evidence when conducting individual-based interventions in controlled settings, it falls short when it comes to assessing structural interventions in relation to policy, the environment, or social system changes [16]. There are other methodologies that are better placed in terms of assessing the health impacts of structural changes, such as sugar taxes, fluoridation policy, and expanding benefits coverage.

Qualitative interviewing techniques, ethnography, focus groups, and participatory action research are crucial to documenting the experience of structural marginalization, how structural factors play out in people's everyday lives, and the social dynamics that influence how communities interact with research and care for their oral health [17,18,20]. A science limited by quantitative clinical research techniques alone cannot properly investigate the social aspects of the issues that it is addressing. Plurality of methods does not constitute weakness in methodology but rather the appropriate scientific approach to complex issues [20].

The same applies to longitudinal and life-course approaches. Social determinants of oral health are complex and dynamic in nature, and negative experiences during childhood exert greater influence on oral health patterns than any other period throughout the lifespan. Moreover, the consequences of prolonged socioeconomic disadvantage cannot be detected in cross-sectional observations no matter how representative the data set may be [20]. Failure to account for these changes means that the significance of structural forces in shaping oral health would remain underestimated and that opportunities for effective prevention would be missed. Establishing longitudinal oral health cohorts is an investment in research capacity development with high potential for equity-focused knowledge generation. 

The global policy enabling environment

The WHO GOHAP and Bangkok Declaration

The development of the WHO Global Oral Health Strategy in 2022 and its subsequent implementation in the GOHAP for the period from 2023 to 2030 is the most significant political decision to promote global oral health equity to date [9,21]. The GOHAP includes a general goal of achieving a 10% decrease in the prevalence of oral conditions by 2030 and involves 100 specific actions, aimed at member states, the WHO Secretariat, international agencies, civil societies, and the private sector [21]. Of all the outlined strategic goals of the Action Plan, three seem to be closest to the research agenda of equity discussed in this paper, namely, the incorporation of oral health in universal health coverage, social and commercial determinants, and the workforce.

The 2024 Bangkok Declaration, signed at the inaugural WHO Global Oral Health Conference and the first United Nations political statement on oral health, gave oral health the designation of global political priority as part of the universal health coverage and Sustainable Development Goal (SDG) discourse [9]. The Declaration recognizes that health without oral health is no health at all, which is both an international policy mandate that authorizes oral health equity as a research issue and a framework of accountability mechanisms at the member state level. As such, this political environment is new to dental researchers because it is an opportunity for them to participate in global policy efforts and assist in the measurement and evaluation of these efforts [9,21]. However, with these political opportunities also come political obligations. Within the GOHAP framework, there are specific measures that must be monitored in relation to equity of oral health and care access. Dental researchers will contribute to meeting these obligations by producing research data for the indicators in question; however, this process can only occur with the use of research methodologies outlined in this paper [12,21].

Universal health coverage and the financing of dental equity

Oral health's omission from universal health coverage benefits packages is a conscious decision rather than an inherent and inescapable aspect of health systems, with profound implications for equity. Where oral health services are not included in universal or public insurance coverage, access depends on individuals' ability to pay, resulting in a system of care provision that reflects and entrenches preexisting social inequities [3]. Research findings are unequivocal; nations that include publicly funded oral health services have significantly lower socioeconomic disparities in oral health service utilization compared to nations without these services [3,20]. That the reorganization of healthcare funding can help alleviate socioeconomic inequities in oral healthcare access is one of the most impactful findings in oral health equity research to date and calls for substantial research funding in health economics, implementation science, and health systems analysis to facilitate implementation [10].

When conducting research to test various models of extending coverage for dental care, equity should be considered as a desired outcome: not only will it be desirable if the extension of coverage results in increased utilization, but it would also be important to see if there was any reduction in the gap between those with more or less advantages [2]. In addition, the way in which implementation affects provider participation, provider distribution, administrative burden, and cultural/linguistic competency should be taken into account. Extended coverage that does not result in access is a failed equity strategy.

Oral-general health integration as an equity strategy

The historical divide between dentistry and general medicine is an example of structural inequity with far-reaching implications. There is commonality in the risk factors for oral diseases, such as sugar intake, smoking, alcohol intake, and psychosocial stress, and other noncommunicable diseases, including a bi-directional relationship between cardiovascular disease, diabetes, adverse pregnancy outcomes, and respiratory problems [1]. The CFR approach was specifically designed to harness this commonality, allowing a holistic approach to health promotion that includes both oral and general health. Nevertheless, institutional barriers to integrating the two fields have prevented this from occurring at the delivery, educational, and research levels [14].

Oral health promotion and basic preventive services within general health programs utilizing community health workers, nurses, and primary care physicians as the first point of contact for oral health interventions can serve as a scalable and equitable strategy for delivering preventive oral healthcare to underserved groups [22]. The research that examines these integrated models needs to be purposely designed within situations of genuine deprivation, by measuring the outcomes with equity stratification, and with adequate follow-up time in order to assess sustainability. Integrated models that can succeed within resource-rich primary care environments might not be replicable in resource-poor environments without modification; research on the issue needs to be done from all angles.

A framework for equity-centered dental research: core principles

It is clear from the existing prevalence of oral diseases throughout the globe along with the disparity of the prevalence rates among different populations that there is a need to have a more equitable research process [23,24]. The following guidelines can be considered as a flexible and universal framework that can be applied by researchers in dentistry to reduce the current levels of health disparity. First, research should be centered on achieving equity as one of its goals. Many times, researchers focus on achieving other outcomes while ignoring equity [25]. In future researches, scientists need to consider ways through which research projects can make a difference to those groups of people who suffer most from oral diseases, yet are still neglected [23].

Just as significant is the requirement for research to go beyond the superficial causal factors of diseases and adopt a structural perspective. The outcomes in terms of oral health do not hinge exclusively on individual choices; they are heavily reliant upon the social and economic context [25]. The income structure, service availability, and environmental settings have to be investigated along with the biological determinants, considering the clear link between the socioeconomic profile and oral health [26]. Another critical area to consider is the influence of communities on research. Those groups that are disproportionately impacted by the lack of equity in oral health cannot be seen only as respondents for the research projects. Instead, they ought to take an active part in research design and implementation [17]. The experience with community-oriented programs indicates that collaboration may lead to more successful and sustainable projects [27].

The third issue to address concerns the appropriateness of research investment relative to disease burden. Oral diseases being one of the most common ailments in the world, research investments and capacities have to match. Research environments in LMICs have to be strengthened, as well as focus on the regions which require attention [28]. Given the significant economic burden posed by untreated oral diseases across the world, this is vital [29].

Moreover, to improve equity in oral healthcare, there needs to be a willingness to embrace different types of research methodologies. Problems of this kind are complex and will require more than a single approach. By using quantitative and qualitative measures and combining them with the analysis made from systems science and health economics, for instance, researchers could get a deeper understanding of the issue [30]. Lastly, it is important to recognize that research, apart from generating information, also involves the process of disseminating such findings. Evidence gathered needs to be communicated effectively in ways other than peer-reviewed journals and used to influence policies, practices, and community efforts.

Integrating policy, innovation, and public health: advancing equity in dental research

There have been growing numbers of studies emphasizing the importance of tackling oral health disparities as part of a larger issue regarding the entire health system and political environment. It is clear that the integration of medicine and dentistry, especially among pediatric patients, represents an example of new approaches based on a more holistic and equity-focused view where oral health issues become an integral component of general health rather than a separate one [31]. In a similar manner, the political aspect can be seen in terms of policy measures, for instance, promoting policies related to the regulation of sugar in the diet and indicating the importance of factors such as nutrition and the commercial aspects when considering oral health issues [32].

However, it is essential to note that the promotion of equity within research entails a focus on accessibility, innovation, and relevance. Studies focused on developing inexpensive and more efficient methods of disinfection, such as those using plants, illustrate the attempts of the scientific community to create applicable innovations which might prove to be especially useful in developing countries [33]. Furthermore, it should be noted that research on the impact of emerging diseases is an important factor in revealing how healthcare inequalities may exacerbate due to disparities in healthcare facilities and prevention strategies [33].

Collectively, these perspectives underscore that equity-centered dental research must strike a balance between global health priorities and locally adaptable innovations, ensuring that both scientific advancements and public health responses remain inclusive, context-sensitive, and accessible to underserved populations.

## Conclusions

Equity should not be viewed as an issue of secondary importance in the field of dentistry. Instead, health equity should form the basis of ethical considerations, scientific reasoning, and practical application of dental knowledge. The causes of inequities in oral health go beyond clinical problems to include social, cultural, economic, and environmental determinants of access to care and equitable participation in dental science research. Despite substantial accomplishments made in terms of biological understandings of the mechanisms underpinning oral diseases, improvements in oral health outcomes among marginalized and vulnerable communities have not been realized.

The reality is that global oral health disparities can be addressed via determinants which are not biologically determined, but rather modifiable. There exists an abundance of theoretical perspectives, strategic approaches, and policy proposals designed to improve oral health equity; yet, they suffer from various limitations and impediments to implementation associated with financial constraints, inadequate infrastructure, lack of personnel, restrictions on policies, and other systemic issues.

In order to produce scientific results that have integrity and respect ethics and have social significance, dental research needs to take into consideration health equity at all stages, from setting the research agenda to data analysis and translating results into practice. Conducting dental research with an equity focus is vital not only for overcoming oral health disparities around the globe but also for ensuring the integrity of dental science itself.
